# Supplementary material for: Integrated TORC1 and PKA signaling control the temporal activation of glucose-induced gene expression in yeast
Source: Nat Commun. 2019 Aug 8;10:3558. doi: 10.1038/s41467-019-11540-y (PMC6687784; doi:10.1038/s41467-019-11540-y)
Supplement: Supplementary file 1 — Supplement information [file 41467_2019_11540_MOESM1_ESM.pdf]

Supplement for Kunkel et. al.

Integrated TORC1 and PKA signaling control the temporal activation of glucose-induced gene expression in yeast

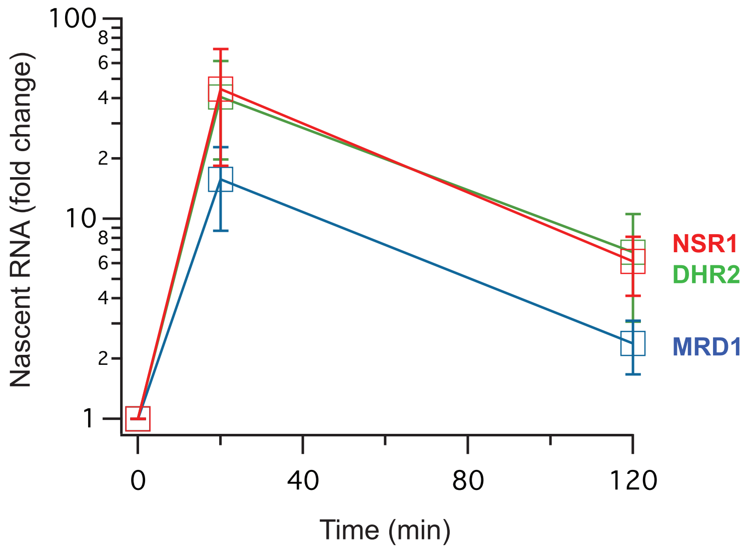

Supplementary Figure 1. Production of new Ribi gene transcripts in glucose. To confirm that the transient increase in Ribi gene mRNA seen in our microarray analysis is due to new transcription, we measured the amount of NSR1, DHR2, and MRD1 RNA bound to RNA Pol II during log growth in glycerol (time = 0), as well as 20 and 120 min after 2% glucose was added to the culture, using qPCR. Each time-point shows the average and standard deviation for three biological replicates, normalized based on the mRNA levels at time=0. In all three cases, there is a dramatic increase in the number of nascent transcripts at the early time point, and then transcription levels drop over time.

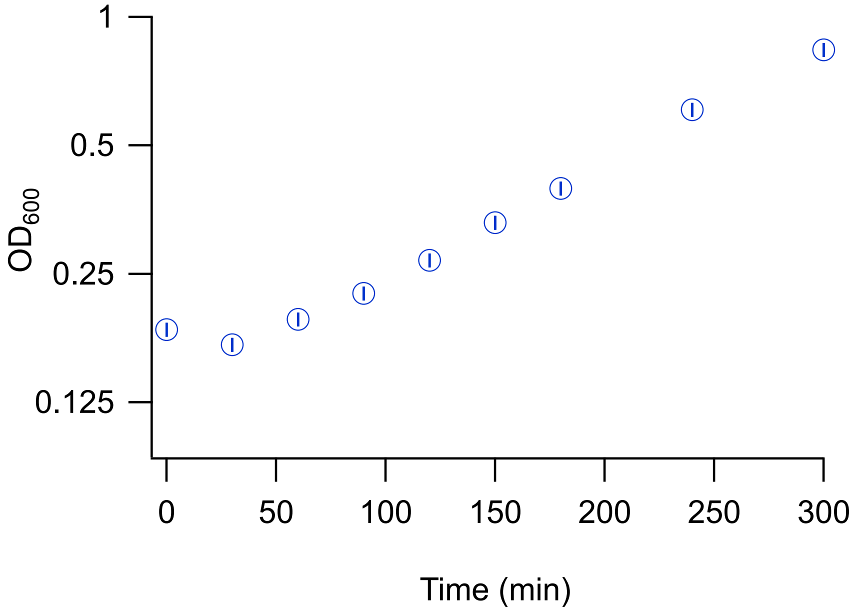

Supplementary Figure 2. Growth curve for Tpk1-3as cells (on a log scale). Tpk1-3as cells were grown to log phase in glycerol, 2% glucose added to the medium (at time = 0) and the optical density (OD_{600}) measured every 30 to 60 min. Each time-point shows the average and standard deviation for three biological replicates (the error bars are smaller than the data points). The cells take less than 30 min to adapt to glucose and enter the log growth phase.

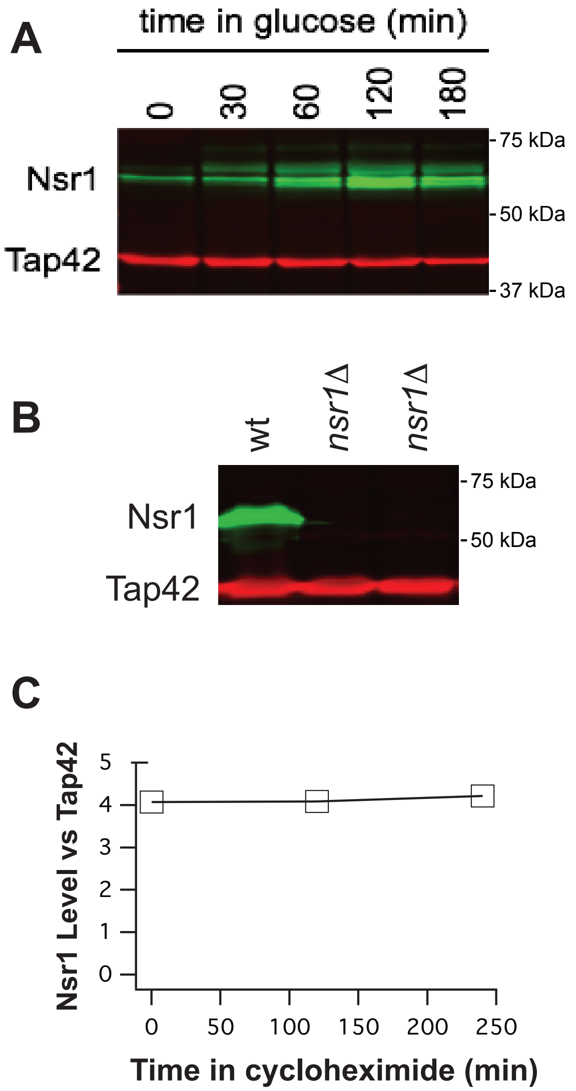

Supplementary Figure 3. Nsr1 protein levels. **(A)** Nsr1 protein levels were measured in cells growing in glycerol (time = 0) and at various time-points after the addition of 2% glucose to the culture using Western blotting. Anti-Tap42 was used as a loading control. **(B)** The Nsr1 antibody used in our analysis is highly specific and does not bind to proteins in the extract from *nsr1Δ* cells. (**C**) Nsr1 levels measured before (time = 0) and after treatment with cycloheximide using a western blot (single experiment).

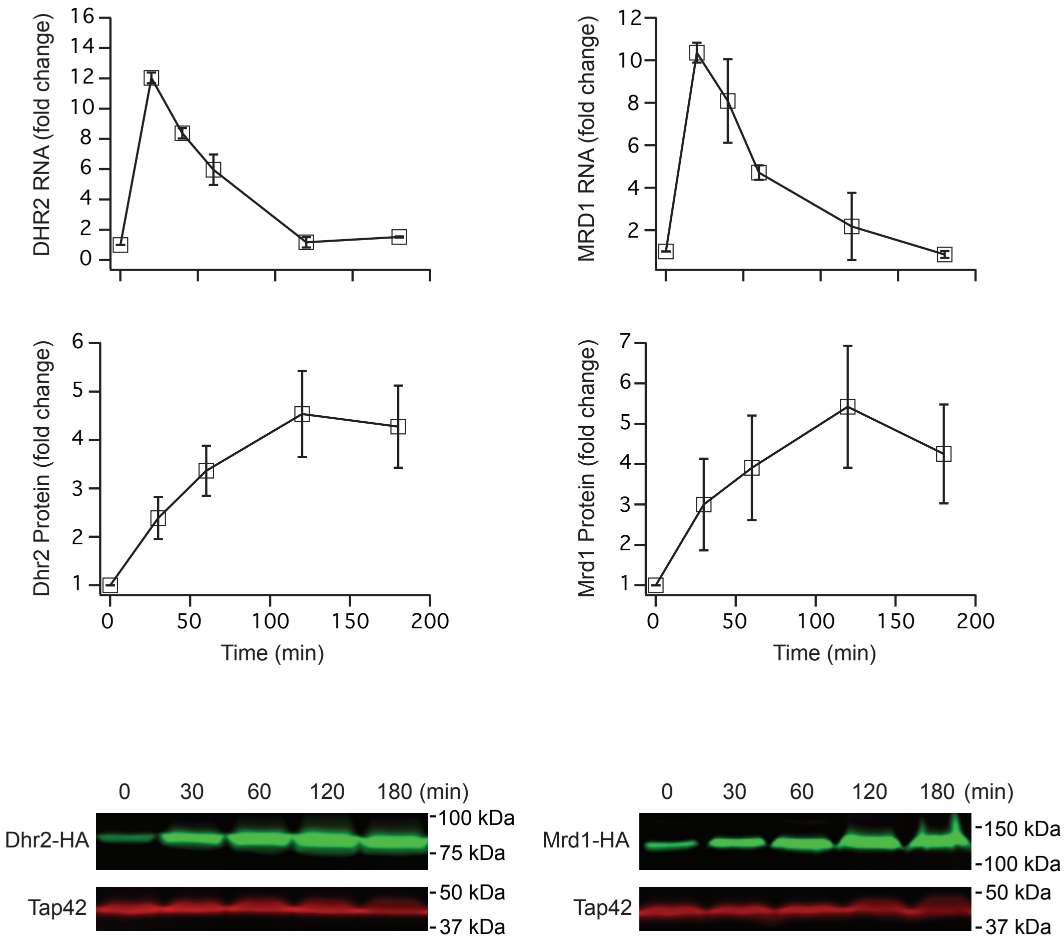

Supplementary Figure 4. Dynamics of Dhr2 and Mrd1 production. The fold change in DHR2 and MRD1 mRNA expression (top panels) and Dhr2 and Mrd1 protein levels (bottom panels) after introduction of glucose to cells growing in glycerol—as measured by qPCR and western blotting. The graphs show the average and standard deviation from three biological replicates, normalized based on the protein or RNA levels at time zero.

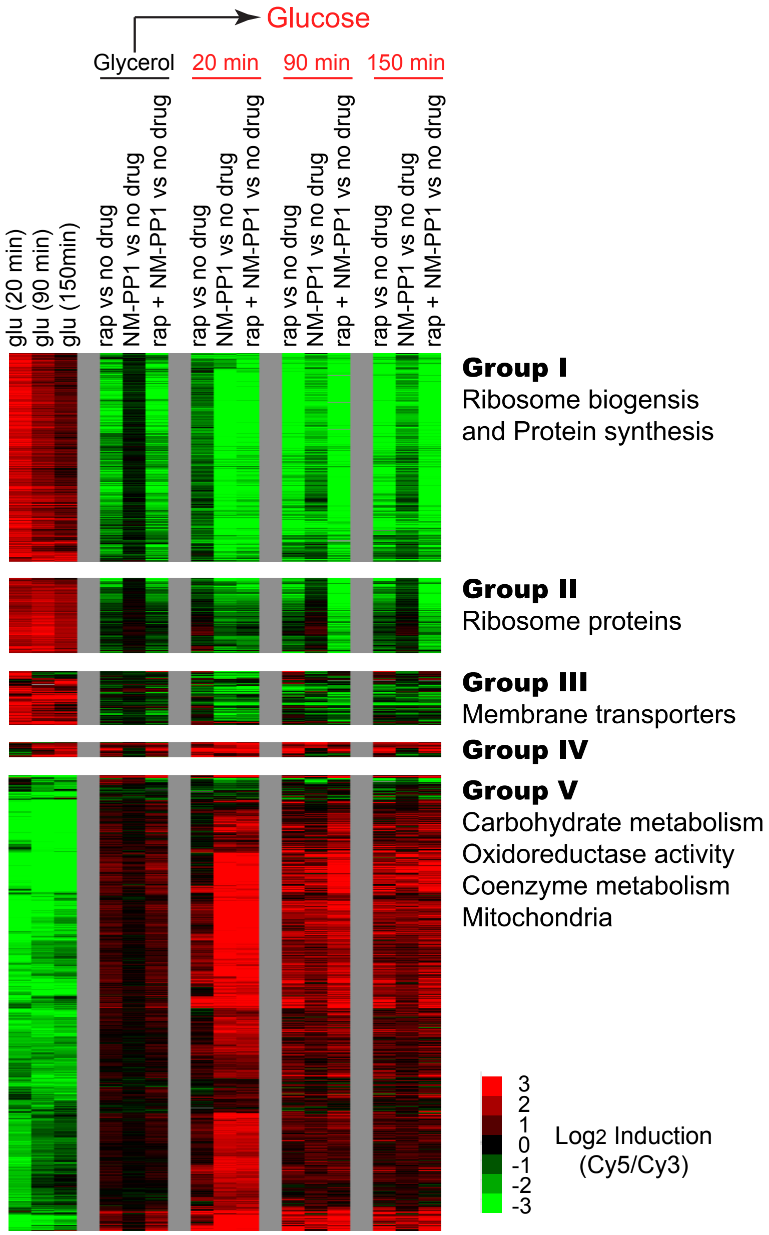

Supplementary Figure 5. TORC1 and PKA signaling. **(Columns 1-3)** The gene expression program activated in glucose was measured by comparing the mRNA from Tpk1-3as cells growing in glycerol (labeled with Cy3) to the mRNA from Tpk1-3as cells exposed to 2% glucose for 20, 90 or 150 min (labeled with Cy5), on a 2-color DNA microarray. **(Columns 4-15)** To quantify the influence that the PKA and TORC1 kinases have on this gene expression program, Tpk1-3as cells (grown as above) were treated with 1-NM-PP1, rapamycin, or rapamycin + 1-NMPP1 for 30 min, leading up to the reported time, and the mRNA levels compared to those from cells treated with DMSO alone, using a two-color microarray. Drug treated samples were labeled with Cy5 while DMSO treated samples were labeled with Cy3. The heat map shows data for all genes with ≥3-fold activation or repression in glucose (Columns 1-3). GO terms are from Fig. 1.

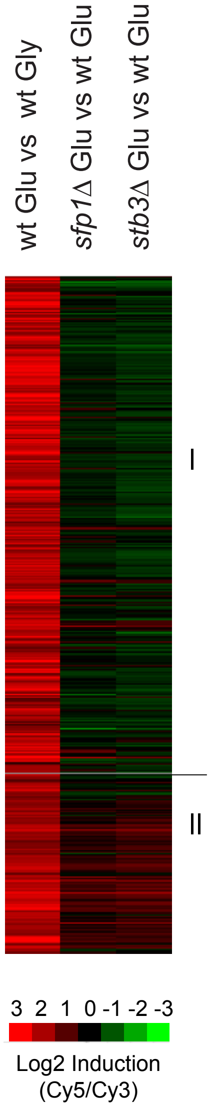

Supplementary Figure 6. Sfp1 and Stb3 signaling during the initial response to glucose repletion. Tpk1-3as cells with Sfp1 or Stb3 deleted were grown to log phase in glycerol, 2% glucose added to the medium, and then snap frozen after 20 min (see Methods). The mRNA isolated from these cells (labeled with Cy5) was then compared to the mRNA from Tpk1-3as cells grown and prepared in an identical way, but labeled with Cy3, to look for defects in the induction of protein and ribosome synthesis (Group I) or ribosome protein (Group II) genes (Columns 2 and 3). The gene induction levels in Tpk1-3as cells grown in glycerol and then exposed to glucose for 20 min (from Figure 1b) are shown in Column I for comparison. The *sfp1*Δ and *stb3*Δ strains have a 1.15 and 1.3-fold decrease in ribosome and protein synthesis gene induction, and a 1.4 and 1.5-fold increase in ribosome protein gene induction, respectively.

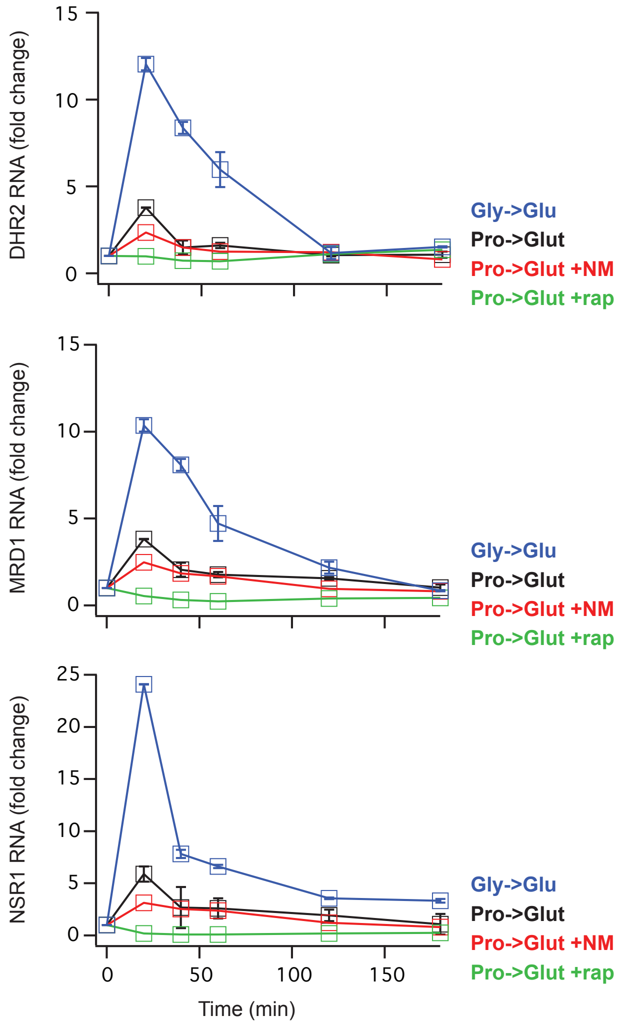

Supplementary Figure 7. Impact of TORC1 activation on Ribi gene expression. To determine if the large pulse of gene expression observed when PKA is activated by the addition of glucose to cells growing in glycerol (blue lines) is also created when TORC1 is activated, we followed the expression of three Ribi genes (NSR1, MRD1 and DHR2) during the transition from growth in synthetic medium with glucose and a low-quality nitrogen source (0.5g/L proline), to growth in synthetic medium with glucose and a high-quality nitrogen source (0.5g/L glutamine) using qPCR. This transition led to a 1.7-fold increase in growth rate but only a small pulse of Ribi gene induction (black lines). Inhibition of PKA with 1-NM-PP1 blocked most of the pulse (red lines)—in line with reports that addition of a nitrogen source to cells triggers weak activation of the PKA pathway (1). However, inhibition of TORC1 with rapamycin (green lines) blocks the entire pulse suggesting that TORC1 is transiently hyper-activated during the proline to glutamine transition. The Gly to Glu and Pro to Glu graphs show the average and standard deviation from three biological replicates, while the Pro to Glut +NM and Pro to Glut +rap data is from a single experiment.

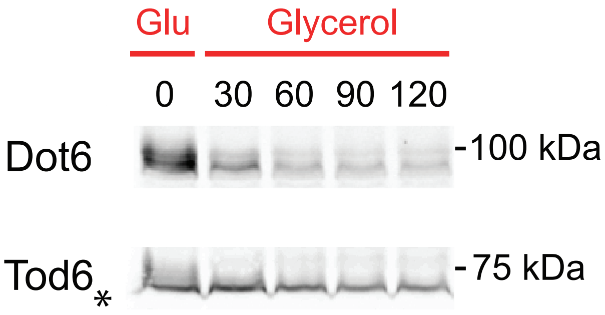

Supplementary Figure 8. Western blot analysis to examine Dot6 and Tod6 protein levels following glucose starvation. Tpk1-3as cells with HA-tagged Dot6 or Tod6 were grown in synthetic complete medium + 2% glucose (as described in the Methods section), and cells harvested before, or at 30 min intervals after, cells were transferred to synthetic complete medium containing 3% glycerol (and no glucose). *The band seen at the bottom of the Tod6 lane is also present in blots examining extracts from wild-type cells (no HA tag) and is therefore not Tod6-HA.

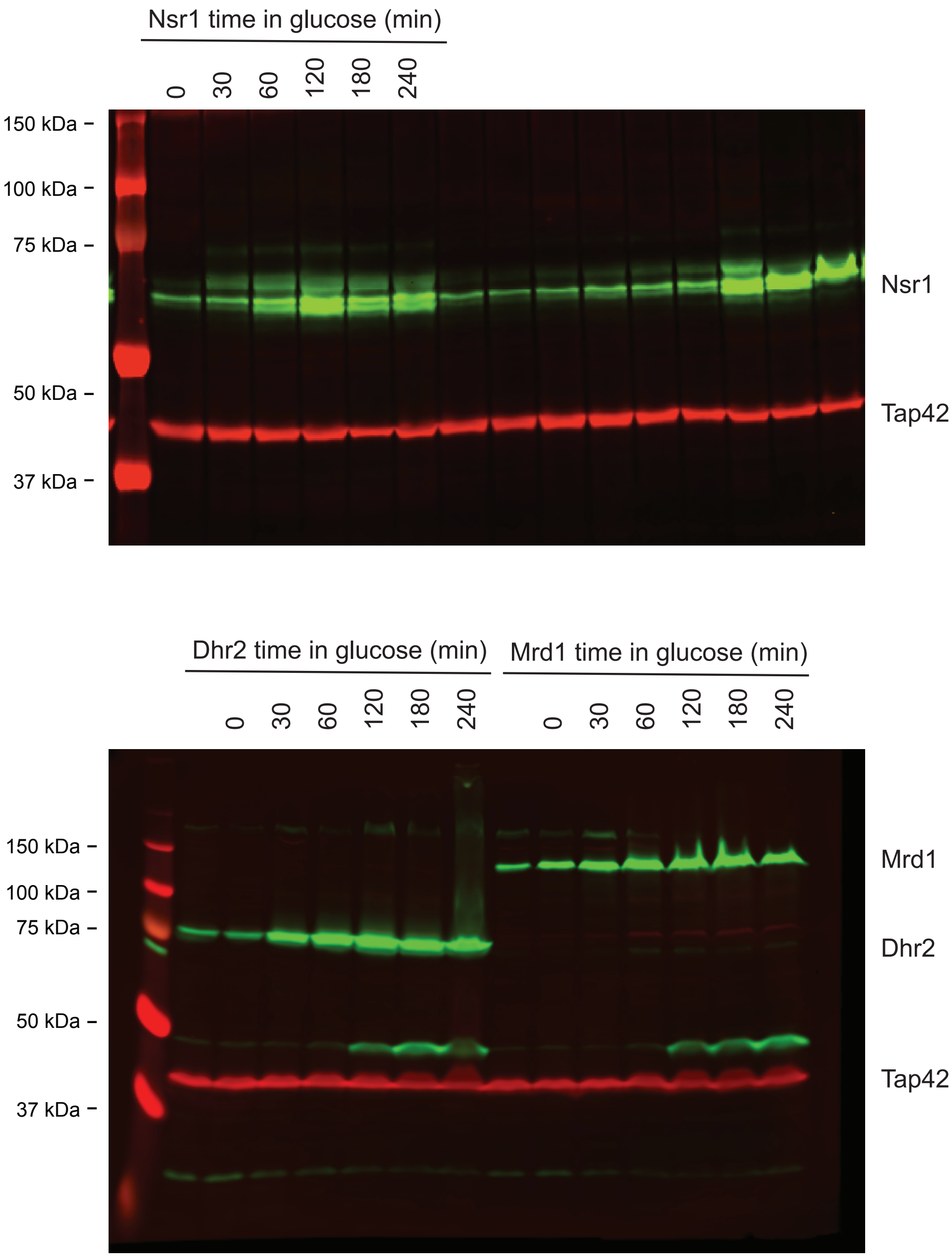

Supplementary Figure 9. Uncropped and unprocessed Western blots examining the level of Nsr1 (upper panel), Dhr2-HA (lower panel) and Mrd1-HA (lower panel) after glucose is added to cells growing in glycerol. The data is the same as that shown in Supplemental Figures 3 and 4.

Supplementary Table 1. List of primers used in this study

Primer#	Primer Name	Sequences 5' to 3'
PR1	TPK1ampf(NotI)	AGCTACCTTGCGGCCGCCAAAGGTAGCACAGAAAGTAGTGTT
PR2	TPK1ampr(BamHI)	ATGCTATAGGATCCGGAGATTCGACTTGTTTGG
PR3	TPK2ampf(NotI)	AGCTACCTTGCGGCCGCCCTCTTGGCCAATTTGTTTT
PR4	TPK2ampr(SalI)	ATGCTATAGTCGACTTAGGGCAACGCTTGTTCTT
PR5	TPK3ampf(NotI)	AGCTACCTTGCGGCCGCAGGCGATCACTCTTTAAGCAA
PR6	TPK3ampr(SalI)	ATGCTATAGTCGACTGTTGTGCAACCTTAGACATCTT
PR79	F2CHK(rev)	ACCCGGGGATCCGTCGACC
PR80	CGCHK(rev)	GGTCATAGCTGTTTCCTGTG
PR101	TPK1seq769F	GGCCAGAAAAACGTGAGAAT
PR104	TPK2seq609F	ACACACAATTCCATATCGAGTTAATA
PR107	TPK3seq722F	GGGATTTCTTCGATGCCATT
PR110	TPK3seq3129R	GGCTGACAATATTCAGCACAA
PR117	TPK1seq3120R	TGCACGAGCTTTCTCTTCCT
PR118	TPK2seq2993R	ACGCACCAACTGCTGATACA
PR127	T7seq	TAATACGACTCACTATAGG
PR128	T3seqrev	AATAACCCTCACTAAAGGG
PR134	TPK1seq1383f	AGGTGGAGCATACCAACGAC
PR135	TPK2seq1370f	CTTGTGGAGCATCCGTTTCT
PR136	TPK3seq1578r	GCGGCATAAAATTTGGCTAC
PR357	Dot6KOf	CCGTGCACGTTCCAGTCTTCCCTCCCTTCTCTGCTCCGTGCACAGGAAACAGCTATGACC
PR358	Dot6KOr	TTTTTTTATTTTTATTTTTTTTTCATTTTAAGTTTTCCCCGTTGTAAAACGACGGCCAGT
PR359	Dot6KOFCP	CCCGTTGTGAAATCTCACC
PR360	Tod6KOf	GAAAAGAATATATCACTGTTCTTATTGAAGTTCCCTCGCGCACAGGAAACAGCTATGACC
PR361	Tod6KOr	TTAAACGAATAGTCAAAACAAACTAAATTCGTCTCTTAGCGTTGTAAAACGACGGCCAGT
PR362	Tod6KOFCP	TTTAGCAACCTCTTTGCAGG
PR390	Stb3KOf	ATTTTGAATAAAAAGACATTATTTCAGACTACCACTAATACACAGGAAACAGCTATGACC2
PR391	Stb3KOr	TACTGTTTTTTTGTTATTTCATGGAACTGGTAAAGAATTCGTTGTAAAACGACGGCCAGT
PR392	Stb3KOFCP	GTAGTAGGCTGATCACTCCGC
PR498	TOD6-FCP	GATTATTTAAAGAGAAAAGGACGTGTAAGTGG
PR499	TOD6-TAPf	TGAACTTCCAGGTATAAATTCTATCTTTAAAAATATATTTGGTCGACGGATCCCCGGGTT
PR500	TOD6-TAPr	TTAAACGAATAGTCAAAACAAACTAAATTCGTCTCTTAGCTCGATGAATTCGAGCTCGTT
PR501	DOT6-FCP	GGCTGCAACCATGAATCGCACCCC
PR502	DOT6-TAPf	TCCGCTGCCAAGCATAAACACTATCTTCAAGGATATGCTGGGTCGACGGATCCCCGGGTT
PR503	DOT6-TAPr	TTTTTTTATTTTTATTTTTTTTTCATTTTAAGTTTTCCCCTCGATGAATTCGAGCTCGTT
PR628	Stb3HAf	ACAATAGTGCTGCGTTTTTATTAATGAGCTTAAAATCTGGTCGACGGATCCCCGGGTT
PR629	Stb3HAr	CTGTTTTTTTGTTATTTCATGGAACTGGTAAAGAATTCTCGATGAATTCGAGCTCGTT
PR630	Stb3HAchk	CGTCACTACAAACCCTAACT
PR3702	MRD1 HA f	AAAGAAACTGGATGTGGATGATGAAGAAAACGAAGGTTTCGGTCGACGGATCCCCGGGTT
PR3703	MRD1 HA r	TAAATTATGAAAGAAAAATACGATAAGCTGATAGGGTAAATCGATGAATTCGAGCTCGTT
PR3704	MDR1 TGCK f	CCAAGGAACAAGCTAATGCC
PR3711	DHR2 HA f	TGCAGCCGCTGTAGCAAAGCAAAAAGTTTCTGATTCAAAAGGTCGACGGATCCCCGGGTT
PR3712	DHR2 HA R	TGCCTTTATATACAATAAAAATGCGCTAAGAGTAGGTGACTCGATGAATTCGAGCTCGTT
PR3713	DHR2 TGCK f	GAAAGAAATGATTGGTGTAA
PR4015	Rpb3 flag F	AATGGGTAATACTGGATCAGGAGGGTATGATAATGCTTGGgggggaggcgggggtgga
PR4016	Rpb3 flag R	GTTCACTTGTTTTTTTTCCTCTATTACGCCCACTTGAGAAgaattcgagctcgtttaaac
PR4017	Rpb3 tag ck	GATCCTCCAAACGAAGGTGA

Supplementary References

1. Donaton MC, *et al.* (2003) The Gap1 general amino acid permease acts as an amino acid sensor for activation of protein kinase A targets in the yeast Saccharomyces cerevisiae. *Mol Microbiol* 50(3):911-929.
