# Supplementary material for: Integrated TORC1 and PKA signaling control the temporal activation of glucose-induced gene expression in yeast
Source: Nat Commun. 2019 Aug 8;10:3558. doi: 10.1038/s41467-019-11540-y (PMC6687784; doi:10.1038/s41467-019-11540-y)
Supplement: Supplementary file 4 — Reporting Summary [file 41467_2019_11540_MOESM4_ESM.pdf]

nature research

Corresponding author(s): Andrew Capaldi

Last updated by author(s): 2019/07/01

Reporting Summary

Nature Research wishes to improve the reproducibility of the work that we publish. This form provides structure for consistency and transparency in reporting. For further information on Nature Research policies, see Authors & Referees and the Editorial Policy Checklist.

Statistics

For all statistical analyses, confirm that the following items are present in the figure legend, table legend, main text, or Methods section.

n/a	Confirmed	
☐	☒	The exact sample size (*n*) for each experimental group/condition, given as a discrete number and unit of measurement
☐	☒	A statement on whether measurements were taken from distinct samples or whether the same sample was measured repeatedly
☐	☒	The statistical test(s) used AND whether they are one- or two-sided<br>*Only common tests should be described solely by name; describe more complex techniques in the Methods section.*
☒	☐	A description of all covariates tested
☒	☐	A description of any assumptions or corrections, such as tests of normality and adjustment for multiple comparisons
☐	☒	A full description of the statistical parameters including central tendency (e.g. means) or other basic estimates (e.g. regression coefficient) AND variation (e.g. standard deviation) or associated estimates of uncertainty (e.g. confidence intervals)
☒	☐	For null hypothesis testing, the test statistic (e.g. *F*, *t*, *r*) with confidence intervals, effect sizes, degrees of freedom and *P* value noted<br>*Give P values as exact values whenever suitable.*
☒	☐	For Bayesian analysis, information on the choice of priors and Markov chain Monte Carlo settings
☒	☐	For hierarchical and complex designs, identification of the appropriate level for tests and full reporting of outcomes
☒	☐	Estimates of effect sizes (e.g. Cohen's *d*, Pearson's *r*), indicating how they were calculated

Our web collection on statistics for biologists contains articles on many of the points above.

Software and code

Policy information about availability of computer code

Data collection	None
Data analysis	GenePix 6.1 software for microarray analysis. Expression component analysis was using simple equations described in the manuscript and run in Excel.

For manuscripts utilizing custom algorithms or software that are central to the research but not yet described in published literature, software must be made available to editors/reviewers. We strongly encourage code deposition in a community repository (e.g. GitHub). See the Nature Research guidelines for submitting code & software for further information.

Data

Policy information about availability of data

All manuscripts must include a data availability statement. This statement should provide the following information, where applicable:

- Accession codes, unique identifiers, or web links for publicly available datasets
- A list of figures that have associated raw data
- A description of any restrictions on data availability

The data supporting the findings of this study are available within the paper and its supplementary information files. The microarrays are now posted in the GEO database and the accession code provided

Field-specific reporting

Please select the one below that is the best fit for your research. If you are not sure, read the appropriate sections before making your selection.

☒ Life sciences ☐ Behavioural & social sciences ☐ Ecological, evolutionary & environmental sciences

For a reference copy of the document with all sections, see nature.com/documents/nr-reporting-summary-flat.pdf

Life sciences study design

All studies must disclose on these points even when the disclosure is negative.

Sample size	Sample size was 1 to 3 biological repeats (3 for all key results), as described in the text. This is standard (or above standard) for these kinds of studies.
Data exclusions	No data was excluded in the analysis described in this study.
Replication	As described under sample size, the data presented here was all repeated 2-3 times, as described in the text of the paper. None of the microarray data collected was excluded from the study. One qPCR experiment was excluded from the paper and analysis because the control samples did not amplify in the expected range. This experiment was rerun and the data included in the paper
Randomization	This is not relevant to this study.
Blinding	The experiments were not done blind (we knew what mutants we were examining), but this knowledge did not influence our analysis.

Reporting for specific materials, systems and methods

We require information from authors about some types of materials, experimental systems and methods used in many studies. Here, indicate whether each material, system or method listed is relevant to your study. If you are not sure if a list item applies to your research, read the appropriate section before selecting a response.

Materials & experimental systems

n/a	Involved in the study	
☐	☒	Antibodies
☐	☒	Eukaryotic cell lines
☒	☐	Palaeontology
☒	☐	Animals and other organisms
☒	☐	Human research participants
☒	☐	Clinical data

Methods

n/a	Involved in the study	
☒	☐	ChIP-seq
☒	☐	Flow cytometry
☒	☐	MRI-based neuroimaging

Antibodies

Antibodies used	anti-HA; Roche 11 867 423 001, anti-Nsr1 from Thermo (MA1-10030), anti-Tap42 from Santa Cruz (sc-67113)
Validation	All primaries were run against a strain missing a tag (anti-HA), or with the target protein knocked out (Nsr1 and Tap42) to show that they are specific.

Eukaryotic cell lines

Policy information about cell lines

Cell line source(s)	All cell lines were created for this paper starting from a standard w303 strain from the O'Shea lab at Harvard (now HHMI)
Authentication	PCR and sequencing was used to confirm all mutations introduced into the yeast cell lines as described in the text.
Mycoplasma contamination	NA
Commonly misidentified lines (See ICLAC register)	NA
